# *FIR/PUF60*: Multifunctional Molecule Through RNA Splicing for Revealing the Novel Disease Mechanism and Effective Individualized Therapies

**DOI:** 10.3390/ijms27020643

**Published:** 2026-01-08

**Authors:** Kazuyuki Matsushita, Kouichi Kitamura, Nobuko Tanaka, Sohei Kobayashi, Yusuke Suenaga, Tyuji Hoshino

**Affiliations:** 1 Department of Laboratory Medicine, Chiba University Hospital, Chiba 260-8677, Japan; 2 Department of Medical Technology and Sciences, Health and Sciences, International University of Health and Welfare, Chiba 286-8686, Japan; 3 Laboratory of Evolutionary Oncology, Chiba Cancer Center Research Institute, Chiba 260-8717, Japan; 4 Department of Molecular Design, Graduate School of Pharmaceutical Sciences, Chiba University, Chiba 260-8675, Japan

**Keywords:** *FIR/PUF60*, RNA splicing, cancer, rare disease, rRNA, mRNA, c-Myc

## Abstract

Disease-specific diversity in RNA transcripts stems from RNA splicing, ribosomal abnormalities, and other factors. However, the mechanisms underlying the regulation of rRNA expression in the nucleolus and mRNA expression in the cytoplasm during cancer and neuronal differentiation remain largely unknown. In this article, we review current knowledge and discuss the regulatory mechanisms of rRNA and mRNA expression in human diseases using the splicing model of PUF60 (poly(U) binding splicing factor 60)—also known as *FUSE-binding protein-interacting repressor (FIR)* (*FUBP1-interacting repressor*), *RoBPI*, *SIAHBP1*, and *VRJS* (Gene ID: 22827). Noncoding RNAs, much like coding RNAs, have been found to be translated into proteins with significant physiological functions. Splicing is also involved in dominant ORF RNAs implicated in the expression of both noncoding and coding RNAs. Here, we analyze recent findings regarding gene splicing, ribosome formation, and the determination of selected ORFs (dominant ORFs) in a system modeled on FIR splicing in two databases (RefSeq and ENSEMBL). rRNA transcription affects ribosomes, whereas mRNA expression and splicing affect the intracellular proteome. Our objective is to develop efficient methods for identifying biomarkers for disease diagnosis and therapeutic targets. In the field of cancer treatment, therapeutic drugs targeting intracellular signaling have proven effective.

## 1. Introduction

**Figure 1:**
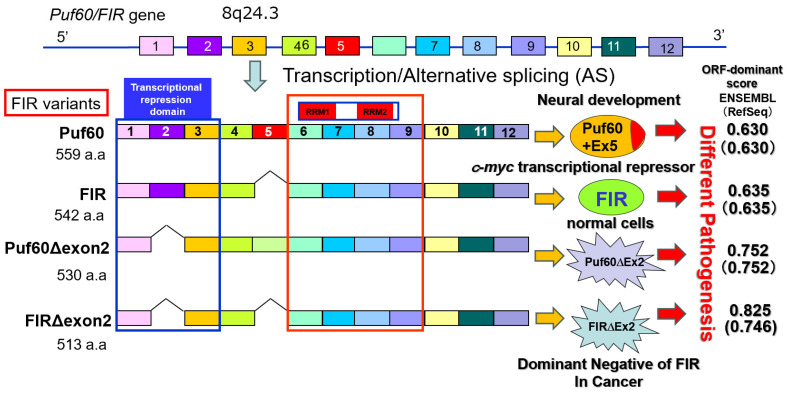
PUF60/FIR and its splicing variants have distinct functions. The *PUF60/FIR* gene exhibits multiple splicing variants, and it is hypothesized that these splicing abnormalities perform different functions depending on the developmental stage and cell type, such as somatic or germ cells. FIRΔexon2, which lacks exon 2, is incapable of repressing *c-Myc* or promoting apoptosis, and was frequently found in human primary cancers, but not in adjacent normal tissues. Multi-omics analysis using human biological samples will be instrumental in investigating the respective functions of each. Different color boxes indicate exons.

The diversity of transcribed RNAs, a hallmark of disease pathology and cellular differentiation, relies heavily on RNA splicing, ribosomes, and other factors. Using FUSE-binding protein-interacting repressor (FIR) (FUBP1-interacting repressor) as a model, we investigated the coupled regulation of rRNA and mRNA expression. Although FIR is a transcriptional repressor of c-Myc, this review focuses on FIR-mediated regulation of rRNA and mRNA expression independent of c-Myc involvement. Elevated c-myc expression has been detected in a wide range of human cancers, indicating a pivotal role for this oncogene in tumor development (Figure 1). It was recently discovered that an interaction between the FIR and the TFIIH/p89/XPB helicase represses c-myc transcription and may be crucial for suppressing tumor formation. In this study, we demonstrate that enforced expression of FIR induces apoptosis. Deletion of the FIR NH2-terminal repression domain (exon2 of FIR) rescued cells from apoptosis, as did the coexpression of c-Myc with FIR; thus, Myc repression mediates FIR-induced apoptosis. Surprisingly, a FIR splice variant, FIRΔexon2, which lacks exon 2, is incapable of repressing *c-Myc* or promoting apoptosis, was frequently found in human primary colorectal cancers, but not in adjacent normal tissues (Figure 1). Coexpression of this splicing variant with repression-competent FIR, in both HeLa cells and the SW480 colon cancer cell line, not only abrogated c-Myc suppression but also inhibited apoptosis. These results strongly suggest that expression of this splicing variant, FIRΔexon2, promotes tumor development by disabling FIR repression, maintaining high levels of c-Myc, and opposing apoptosis in colorectal cancer [1].

This review focuses on FIR, a transcriptional repressor of the *c-myc* gene. FIR is a unique gene in which somatic splicing abnormalities contribute to oncogenesis, while germline variants contribute to neurodevelopmental delay. Somatic splicing abnormalities of FIR (specifically the expression of FIRΔexon2) induce c-myc expression and lead to oncogenesis [1]. Meanwhile, the splicing expression ratio (FIR/PUF60 (FIR + exon5)) has been shown to be important for neuronal differentiation [2]. Furthermore, pathogenic germline variants of this gene have been reported to cause neurodevelopmental disorders, such as Verheij syndrome [3,4]. We present a literature review to explore why FIR splicing is involved in such diverse functions. Our approach also addresses the crosstalk between FIR splicing and the transcriptional regulation of mRNA and rRNA, as this regulation is believed to be dysregulated in both cancer and neurodevelopmental disorders [5]. Thus, it has been suggested that FIR may be involved in the synergy between rRNA and mRNA [5].

**Figure 2:**
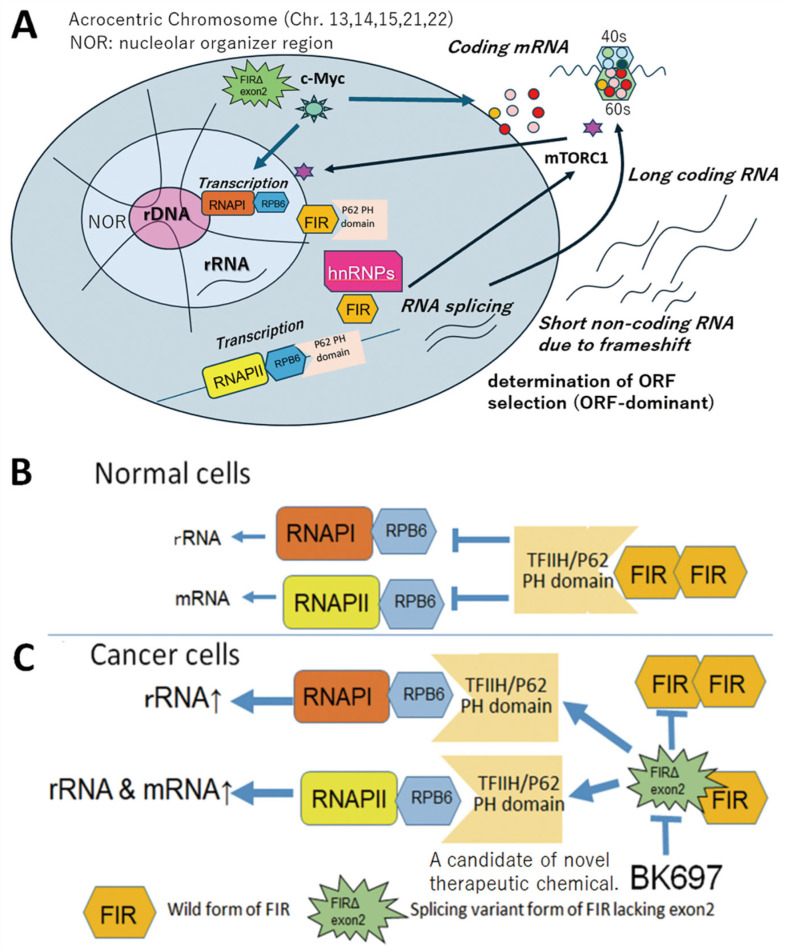
RNA splicing, ribosome formation, and determination of ORF selection (ORF-dominant) in a FIR splicing model. (**A**) rRNA is transcribed from ribosomal DNA (rDNA) or rRNA genes in the nucleolus, and the RPs that constitute the ribosome are generated. The pleckstrin homology (P62 PH) subunit of TFIIH interacts with RPB6, a common amino-terminal tail (NTT) of RNAPI, RNAPII, and RNAPIII. The short arms of acrocentric chromosomes (13, 14, 15, 21, and 22) contain 47S rDNA organized into tandem repeat arrays. In human diseases, fusion between these acrocentric chromosomes results in Robertsonian translocations (RTs) involving the loss of the short arms containing 47S rDNA. In RTs, insufficient rRNA synthesis and RiBi deficiency induce “ribosomopathies,” which are typically associated with malignant tumors. The short arms of acrocentric chromosomes (13, 14, 15, 21, and 22) contain 47S rDNA organized into tandem repeat arrays. (**B**) FIRΔexon2 inhibits P62-RPB6/RNAPI/II interaction for rRNA and mRNA activation. In non-cancer conditions, FIR interferes P62 to bind the RPB6 of RNAPI and RNAPII. (**C**) FIRΔexon2 drives and accelerates rRNA gene transcription in the nucleolus. The RNAPI to RNAPII switching by FIRΔexon2 leads to atypical rRNA transcription (upward arrow). BK697 targeted FIRΔexon2 suppression. These results elucidated the novel mechanism indicating that FIRΔexon2 regulates dynamic rRNA and mRNA expression (upward arrow) is a potential therapeutic target for cancer (modified Figure 7 of Reference [5]).

The far-upstream element (FUSE) is a sequence required for the proper expression of the human *c-myc* gene, and the FUSE-binding protein (FUBP) 1 transcriptionally activates *c-myc* [6]. Yeast two-hybrid analysis revealed that FUBP1 binds to a protein exhibiting transcriptional inhibitory activity, designated FUBP1-interacting repressor (FIR) [7]. The aim of this study was to elucidate the combined mechanism of histone modification, transcription, alternative splicing, and RiBi mediated by FIR—a splicing variant of PUF60 (poly(U)-binding splicing factor 60) lacking exon 5 [6,8]—and its splicing variant. FIR and PUF60 are single-strand (ss) nucleic acid (DNA/RNA) binding proteins; FIR is a transcriptional repressor of the *c-myc* gene [6], whereas PUF60 is a splicing factor [8]. FIRΔexon2 is overexpressed in cancers as a dominant-negative form of FIR [1,9,10,11,12,13]. The regulation of mRNA and ribosomal RNA (rRNA) transcription, ribosomal protein (RP) synthesis, and ribosome biogenesis (RiBi) is essential for cell survival [14] (Figure 1 and Figure 2). The pleckstrin homology (PH) subunit p62 of TFIIH interacts with RPB6, a common amino-terminal tail (NTT) shared by RNAPI and RNAPII [15,16] (Figure 2B,C). Nucleolar RNAPI directly activates rRNA gene transcription under normal conditions; however, RNAPII becomes involved in both rRNA and mRNA transcription under atypical conditions [17] (Figure 2A). Ribosomes are composed of large (60S) and small (40S) subunits, forming a complex of proteins and RNAs [18]. The small 40S ribosomal subunit contains one rRNA (18S) and 33 RPs, whereas the large 60S subunit contains three rRNAs (28S, 5.8S, and 5S) and 47 RPs [19]. The short arms of acrocentric chromosomes (13, 14, 15, 21, and 22) contain 47S rDNA organized into tandem repeat clusters [20] (Figure 2A). In Robertsonian translocations, insufficient rRNA synthesis and impaired RiBi induce “ribosomopathies,” which are often accompanied by malignant tumors [20,21] (Figure 2A).

Furthermore, RNA transcripts contain multiple ORFs, and the selection of a dominant ORF determines whether the RNA functions as a noncoding RNA or is translated into protein, ultimately impacting disease pathology (dominant ORF) [22,23]. There are also several reports of RNAs previously classified as noncoding that are translated into proteins and have physiological functions. Splicing is also believed to be involved in ORF dominance regarding the expression of noncoding and coding RNAs. The transcribed RNA contains multiple ORFs, and the selection of the dominant ORF determines the expression of noncoding or coding RNAs—which are translated into proteins—and ultimately influences disease pathology (dominant ORF). Splicing is also believed to play a role in the dominant ORF RNAs involved in the expression of noncoding and coding RNAs. In this article, we review recent findings on gene splicing, ribosome biogenesis, and the determination of selected ORFs (ORF-dominance) in a system modeled on FIR splicing (Table S1, Figure 1 and Figure 2A). When FIR transcripts were analyzed using ENSEMBL and RefSeq data, the ORF dominance scores were as follows: PUF60 (ENSEMBL 0.630/RefSeq 0.630), PUF60Δexon2 (0.752/0.752), FIR (0.635/0.635), and FIRΔexon2 (0.825/0.746).

## 2. Change in PUF60 Molecular Function by Alternative Splicing as an RNA Splicing Model

### 2.1. Transcriptional Regulation with Splicing Activity by a Single-Strand Nucleic Acid Binding Protein

**Figure 3:**
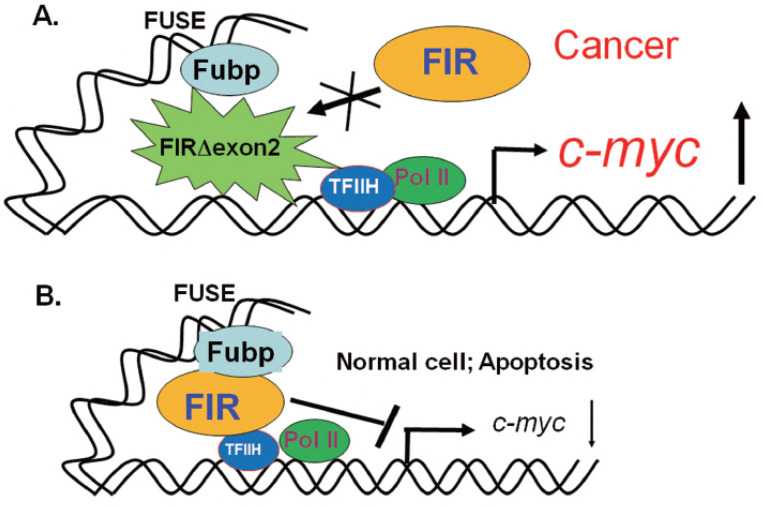
FIR splicing in somatic cells contributes to oncogenesis via c-myc transcriptional activation. Mechanism of dominant interference between FIR and FIRΔexon2. *FIR* splicing in somatic cells contributes to oncogenesis via *c-myc* transcriptional activation. (**A**) In cancer, the FIRΔexon2 splicing variant—in which the FIR exon 2 transcriptional repression site is deleted—is expressed, and its dominant-negative effect increases *c-myc* transcription (large upward arrow). I would like to share an experience where interacting with patient groups helped sustain my motivation for research. (**B**) In normal cells, FIR (FUBP1-interacting repressor) suppresses the transcriptional activator FUBP1, which binds upstream of the *c-myc* promoter. As shown at the bottom of the slide, in normal cells, FIR suppresses the transcriptional activator FUBP, which binds upstream of the c-myc promoter (small downward arrow). Conversely, in malignant lymphomas and solid cancers, as shown at the top of the slide, the FIRΔexon2 splicing variant—which lacks the FIR exon 2 transcriptional repression site—is expressed. It has been reported that the resulting dominant-negative effect upregulates c-myc transcription [1].

FIR functions as a transcriptional repressor of the *c-myc* gene [6,7]. FIRΔexon2 activates c-myc transcription via a dominant-negative effect on FIR [1] (Figure 1). The FIR family (FIR, PUF60, and FIRΔexon2) contains three RRMs (RNA recognition motifs; RRM1, RRM2, and RRM3). RRM3 (a U2AF homology motif; UHM) binds directly to the splicing factor SF3B1 (SAP155) [24]. FIR and FIRΔexon2 were coimmunoprecipitated with SF3B1, and SF3B1 is required for the splicing of FIR pre-mRNA [25]. RRM1 binds to the *c-myc* gene promoter, while RRM2 binds to a transcriptional activator [7,26,27]. FIR (PUF60) interacts with splicing regulatory proteins (hnRNPs), is involved in splicing, and plays a role in neuronal differentiation [8,28] (Figure 2B,C). FIR (PUF60) is a nucleic acid-binding protein involved in pre-mRNA splicing and transcriptional regulation. It promotes the splicing of introns with weak 3′ splice sites by binding to the polypyrimidine tract and cooperating with U2AF65 (U2AF2). PUF60 contains two RNA recognition motifs (RRMs) and a U2 homology motif (UHM), which facilitates interactions with splicing factors such as U2AF2, SF1, and SF3B1. In addition to splicing, PUF60 acts as a transcriptional repressor of MYC by forming a complex with FUBP1 and TFIIH, thereby influencing cell proliferation and apoptosis (Figure 3).

### 2.2. DNA-Damage Repair and Cell Cycle Control by PUF60

Alternative splicing of PUF60/FIR is involved in DNA damage repair [29,30]. The association between alternative splicing and cancer pathogenesis is linked to the potential relationship between alternative splicing, DNA damage, and gastrointestinal cancers. Alternative splicing leads to genetic instability, which is considered a driving force for tumorigenesis.

FIR and FIRΔexon2 form homo- or heterodimers that complex with SF3B1 (SAP155). SF3B1, a subunit of the essential splicing factor 3b subcomplex within the spliceosome, is required for proper splicing of P27Kip1 pre-mRNA, and P27Kip1 arrests cells in G1 [30]. Conversely, FIR was coimmunoprecipitated with Ku86 and DNA-PKcs. siRNA targeting Ku86/Ku70 reduced FIR and P27Kip1 expression, whereas siRNA targeting FIR reduced Ku86/XRCC5 and P27Kip1 expression [30]. Thus, the mechanistic interaction of FIR/FIRΔexon2/SF3B1 bridges c-myc expression and P27Kip1, potentially integrating cell cycle progression and c-myc transcription within the cell. Bleomycin (BLM) is an anticancer agent that induces DNA breaks. Taken together, the FIR/SF3B1 interaction modulates FIR splicing and is involved in cell cycle control or cell fate via P27Kip1 and c-myc in the BLM-induced DNA damage pathway [30].

The FIR adenovirus vector (Ad-FIR) is involved in the DNA damage repair response induced by BLM and is therefore applicable to other DNA-damaging agents. To examine the effect of Ad-FIR on DNA damage repair, BLM, X-rays, and carbon-ion irradiation were used as DNA-damaging agents. Ad-FIR enhanced BLM-induced DNA damage, as indicated by γH2AX in vitro. BLM treatment increased endogenous nuclear FIR expression in TE-2 cells, and P27Kip1 expression was suppressed by TP53 siRNA and BLM treatment. Furthermore, Ad-FIRΔexon2 transcriptional repression domain decreased Ku86 expression. The combination of Ad-FIR and BLM in the presence of TP53 siRNA increased DNA damage [31]. FBW7 expression was significantly decreased in esophageal squamous cell carcinoma (ESCC) [13]. FIR and FIRΔexon2 were overexpressed in ESCC [13]. Moreover, anticancer drugs (cis-diamminedichloroplatinum/cisplatin [CDDP] or 5-fluorouracil [5-FU]) and FIR knockdown by small interfering RNA (siRNA) increased Cyclin E, whereas siRNA-mediated knockdown of FIRΔexon2 decreased Cyclin E expression in ESCC cell lines (TE1, TE2, and T.Tn). Knockdown of SF3B1, a splicing factor required for proper alternative splicing of FIR pre-mRNA, decreased Cyclin E. Three-dimensional structural analysis revealed a hypothetical mechanism for the inhibition of FBW7 function by FIR/FIRΔexon2, representing a novel mechanism of Cyclin E overexpression via the FIR/FIRΔexon2-FBW7 interaction. Elevated Cyclin E expression was induced, in part, by the potential FIR/FIRΔexon2-FBW7 interaction in ESCC [13].

### 2.3. Effects on the EIF/mTORC1/p70S6K Pathway

This study demonstrated that FIR contains a highly conserved acidic string region, similar to that of the RPB6 subunit of RNAPI. X-ray crystal structures of the three FIR domains, RRM1, RRM2, and UHM, were obtained using protein data base (PDB: https://www.rcsb.org/, 4 January 2026) codes 2QFJ, 2KXH, and 3DXB, respectively. siRNA-mediated knockdown of FIR suppressed P62 expression in vivo in neural and cancer cells. Isothermal titration calorimetry (ITC) measurements revealed a molecular interaction between authentic FIR, rather than FIRΔexon2, and P62 [5]. siRNA knockdown of FIR and FIRΔexon2 led to dynamic changes in rRNA expression as assessed by RNA-seq analysis. qRT-PCR analysis confirmed that FIR and FIRΔexon2 significantly altered the expression of various rRNAs on specific chromosomes [5]. Furthermore, FIR and FIRΔexon2 affected the eukaryotic initiation factor (EIF)/mechanistic target of rapamycin (mTOR)/RP S6 kinase (p70S6K) signaling pathway, based on comparisons with the Gene Expression Omnibus database. FIR and FIRΔexon2 are bona fide dynamic modulators of rRNA genes via interaction with the P62 subunit of TFIIH [5]. As RPs and the EIF/mTOR/p70S6K pathway are essential for neural and tumor development, FIR and FIRΔexon2 provide new insights into cell proliferation, neural development, or ribosomopathies by stimulating dynamic ribosome biosynthesis.

### 2.4. Ribosome Biogenesis

The FIR and FIRΔexon2 contribute to the biosynthesis of numerous RPs via transcriptional and posttranscriptional regulation of RP genes on specific chromosomes. Several RPs were coimmunoprecipitated with FIR and FIRΔexon2 proteins in vivo, and RNA sequencing (RNA-seq) combined with chromatin immunoprecipitation (ChIP) analyses revealed that these proteins coordinate histone modification and alternative splicing [5]. Altered expression of FIR or FIRΔexon2 affected endogenous mRNA expression, including that of *PUF60* and *SLC3A2*. The *SLC3A2* and *c-Myc* genes are regulated by the long noncoding RNA SNHG1, suggesting that the FIR family partially activates RP biosynthesis via *c-Myc*. A recent study reported that the *SLC3A2* and *c-Myc* genes are regulated by the long noncoding RNA *SNHG1* [32]. RPs comprise large and small subunits (e.g., 60S and 40S), form a protein-RNA complex, synthesize proteins in vivo and are associated with posttranscriptional regulation or translational modification [18,33,34,35]. Ribosomal RNAs (rRNAs) are transcribed from ribosomal DNA (rDNA) by RNA polymerase I (Pol I) in the nucleolus, and the RPs constituting the ribosome are generated. The transcription factor MYC enhances Pol I activation by binding to the rDNA promoter [36], and RPs are associated with ribosome biogenesis (RiBi), DNA damage repair, apoptosis, cell proliferation, cell differentiation, and alternative splicing [37,38]. In cancer, RP gene expression is upregulated, RP production is altered, apoptosis is inhibited, and cell cycle arrest is induced [39,40,41,42,43]. Ribosomal RNA (rRNA) transcription, RP synthesis, and ribosome biogenesis (RiBi) must be tightly regulated and controlled via a combined feedback mechanism to ensure normal cell survival. Human transcription factor IIH (TFIIH) is an essential factor with a seven-subunit core, including the P62 subunit, and is critical for DNA repair and transcription. Three RNAPs—RNAPI, II, and III—possess a common subunit, RPB6, which interacts with p62 of TFIIH. The FUBP1-interacting repressor (FIR) acts as a *c-myc* transcription suppressor by inhibiting the DNA helicase activity of TFIIH. FIR and PUF60 are single-stranded nucleic acid (DNA/RNA) binding proteins; P62 and P89, subunits of the TFIIH complex, have been found to consistently coimmunoprecipitate with FIR.

## 3. Splicing of PUF60/FIR Regulates mRNA and rRNA Expression

### 3.1. Regulation of rDNA Expression by RNA-Dependent Chromatin Regulation and Acrocentric Chromosome Assembly

The RPs constituting the ribosomes were generated from rRNAs transcribed from rDNA by RNAPI in the nucleolus [5]. Overlap of FIR and FIRΔexon2 was observed on chromosomes 17, 12, 19, and 1. Human rDNAs have been mapped primarily to acrocentric chromosomes 13, 14, 15, 21, and 22 [20]. Further research is required to reveal how FIR and FIRΔexon2 were involved in the transcription of various nucleolar rRNAs and nuclear mRNAs on specific chromosomes (Figure 2A).

### 3.2. Crosstalk Between mRNA and rRNA Expression at Transcription Level

**Figure 4:**
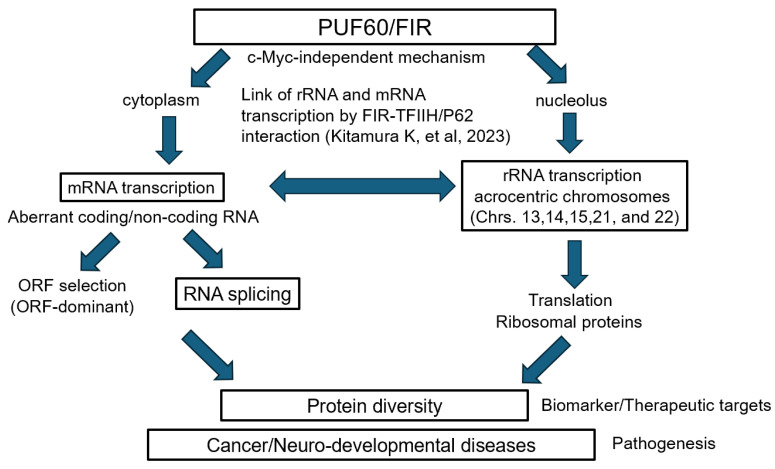
FIR splicing is involved in various functions in a c-Myc-independent manner. Crosstalk between FIR splicing and the transcriptional regulation of mRNA and rRNA. Transcriptional regulation of mRNA and rRNA is dysregulated in cancer and neurodevelopmental disorders. It has been reported that FIR may be involved in the synergy between rRNA and mRNA [5]. Furthermore, transcribed RNA contains multiple ORFs; the selection of a dominant ORF determines the expression of noncoding or coding RNAs (which are translated into proteins) and, ultimately, affects disease pathology (ORF-dominance). Splicing is also implicated in ORF-dominant RNAs involved in the expression of noncoding and coding RNAs.

The FIR family comprises splicing variants (FIR, PUF60, PUF60Δexon2, and FIRΔexon2) and contains three RRMs: RRM1, RRM2, and RRM3 (U2AF homology motif, UHM) (Figure 1) [24,27]. The former study demonstrates the importance of the coupling between rRNA and mRNA transcription in cancer regarding the FIR-TFIIH/P62 interaction [5] (Figure 2A and Figure 4). A novel common pathway of nucleolar rRNA and mRNA transcription mediated by FIR/FIRΔexon2 is proposed in cancer as well as in intractable diseases. The regulation of rRNA expression by FIR and FIRΔexon2 supports the concept that human congenital “ribosomopathies” undergo a paradoxical transition from early symptoms caused by cellular hypoproliferation to an elevated cancer risk later in life [44]. In noncancerous conditions, FIR represses c-myc transcription; consequently, rRNA and mRNA transcription is regulated at physiological levels associated with low c-myc expression [36,45]. In cancer, FIRΔexon2 inhibits the FIR-P62 interaction [5]. This potentially triggers P62 to interact with the RPB6 subunit of RNAPI/II instead of FIR, thereby activating rRNA and mRNA transcription [5]. This study proposes that this c-myc-independent mechanism by FIRΔexon2 partly contributes to the upregulation of rRNA and mRNA transcription in cancer cells. However, c-myc-independent role of FIRΔexon2 in regulating rRNA/mRNA transcription is intriguing but not always clearly separated from c-myc-dependent mechanisms.

### 3.3. Chromatin-Histone Remodeling

Brahma-related gene 1 (BRG1), an ATPase subunit of the SWItch/sucrose nonfermentable (SWI/SNF) chromatin remodeling complex, controls multipotent neural crest formation by regulating epithelial–mesenchymal transition (EMT)-related genes in conjunction with chromodomain-helicase-DNA-binding protein 7 (CHD7). CHD7 is responsible for CHARGE syndrome. Mice with a complete FIR knockout (FIR^−/−^) exhibited embryonic lethality prior to E9.5, suggesting that FIR is pivotal for survival and crucial for development [12]. FIRΔexon2 acetylated H3K27 at the BRG1 promoter, as determined by ChIP, and suppressed the posttranscriptional expression of BRG1. FIRΔexon2 mRNA levels were elevated in human gastric cancers but not in noninvasive gastric tumors in FIR hetero-knockout mice (FIR^+/−^). FIRΔexon2 participates in the multi-step posttranscriptional regulation of BRG1, affecting epithelial–mesenchymal transition (EMT) via the BRG1/Snai1/E-cadherin pathway and promoting tumor proliferation and invasion in gastric cancers [46]. Chromatin-histone remodeling, transcription, and alternative splicing are cooperatively mediated to activate or repress target genes during various cellular processes [45,47,48,49,50]. MYC regulates protein synthesis by stimulating ribosomal proteins (RPS and RPL; small and large ribosomal subunits, respectively [45]), leading to the failure of the mitotic translational switch [51]. MYC plays important roles in cell growth and cancer regulatory networks [52,53,54], as well as in longevity and health [55] through the regulation of ribosome biosynthesis (RiBi). Furthermore, impaired RiBi complexes control TP53 protein levels in response to MYC [56]. Overexpression of FIR or FIRΔexon2 altered H3K27 acetylation at the promoters of the *FIR (PUF60)* gene, as well as several other genes, indicating autocatalytic regulation of *FIR (PUF60)*. Together, FIR and FIRΔexon2 perform distinct functions in the *FIR (PUF60)* autocatalytic feedback loop and in the regulation of the *SLC3A2* gene, which are essential for RP synthesis in carcinogenesis.

## 4. Functions of FIR/PUF60 in Human Diseases

### 4.1. Cancer

PUF60 overexpression is observed in many cancer types (e.g., breast, bladder, leukemia) and correlates with a poor prognosis. It regulates the alternative splicing of oncogenes and tumor suppressor genes. For instance, PUF60 deletion alters CDC25 isoforms, causing cell cycle arrest at the G2/M phase in tumor cells [57]. C57BL6 mice heterozygous for FIR (FIR^+/−^) were generated. Complete FIR deletion (FIR^−/−^) proved lethal to the embryo prior to E9.5; therefore, it is essential for embryogenesis [12]. This strongly suggests that FIR insufficiency is crucial for carcinogenesis [12]. FIR^+/−^ mice exhibited prominent upregulation of c-myc mRNA, particularly in peripheral blood, without any significant pathogenic phenotype. Furthermore, elevated expression of FIRΔexon2/FIR mRNA was detected in human leukemia samples and cell lines. FIR^+/−^TP53^−/−^ mice developed T-cell acute lymphoblastic leukemia (T-ALL) with increased organ or bone marrow invasion and a poor prognosis [12]. The alternative splicing of FIR, which generates FIRΔexon2, may contribute to both colorectal carcinogenesis and leukemogenesis [12].

**Figure 5:**
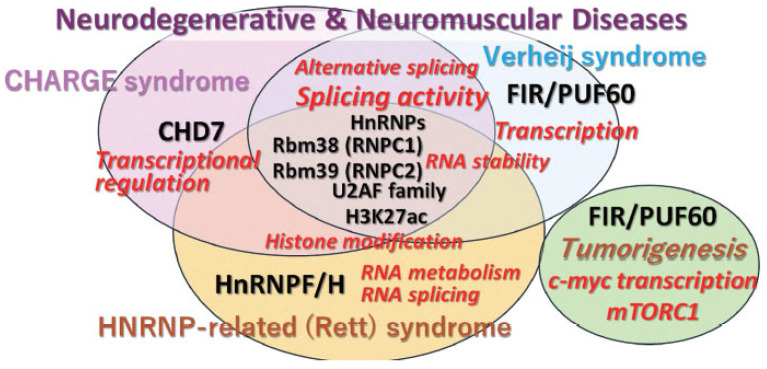
Multifunctional Puf60/FIR mechanism partly responsible for the phenotypic overlap between Verheij syndrome, CHARGE syndrome, and hnRNP-related disorders (Rett). The factors and functions associated with these disorders are listed on this slide. Differential diagnosis is required among these related neurodegenerative and neuromuscular conditions. Different color indicated the categories of the diseases.

Regarding the association between FIR/PUF60 and cancer, there are several reports concerning various types of cancer, including lung cancer [57,58], breast cancer [59,60,61,62], ovarian cancer [63], esophageal cancer [64], gastric cancer [44,65,66,67,68], colorectal cancer [1,10,69], hepatocellular carcinoma [29,30], renal cancer [70], bladder cancer [71], and brain tumors (glioma) [72] (Figure 5).

### 4.2. Neurodevelopmental Disorders

**Figure 6:**
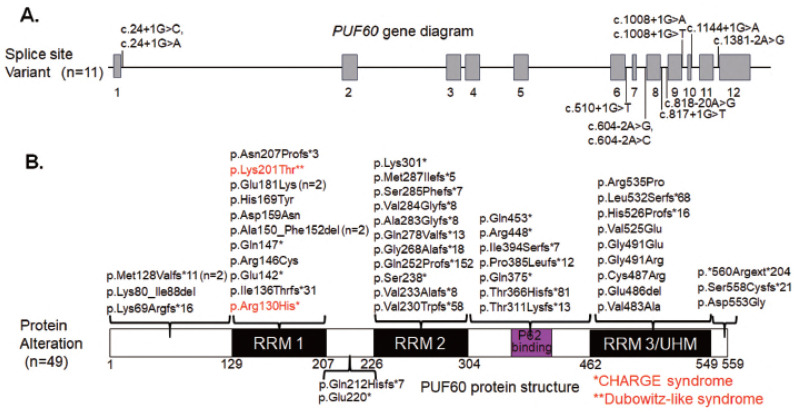
Recent reports of rare disease patients with pathogenic variants in the *FIR/PUF60* region. Verheij syndrome and CHARGE syndrome, patients with congenital anomalies carrying *FIR/PUF60* mutations were summarized from 2009 to 2025. (**A**) Pathogenic variants in *FIR/PUF60* that affect splining. (**B**) Mutations are found in the RRM1, RRM2, and RRM3/UHM regions, with RRM1 being particularly common, although its function remains unknown. Verheij syndrome; 66 cases, CHARGE syndrome; 1 case, Dubowitz-like syndrome; 1 case, and unknown disease; 1 case. Verheij syndrome caused by *PUF60* gene were missense mutations; 14 cases, nonsense mutations; 8 cases, frameshift mutations; 22 cases, splicing abnormalities; 11 cases, 8q24 deletions (13.1 kb~8.35 Mbp) including *PUF60*; 8 cases, 8q24 duplications including *PUF60*; 1 case, and 5 other cases (4 deletions, 1 extension). Germline mutations are common in RRMs, but they were accumulated in no site-specific sites considered as loss-of-function mutations. This suggests that PUF60 plays an important role in neurodevelopment and cell differentiation. Age of onset: 3 cases between 22 and 28 weeks of fetal life, 64 cases between 4 months and 48 years of age (mostly in children), and 2 cases of unknown age. Black asterisks indicated stop codon.

Human germline genetic abnormalities in FIR (PUF60) are believed to underlie Verheij syndrome [73], CHARGE syndrome [74], and HnRNP disorders [75]. In embryonic stem cells, the expression ratio of FIR and PUF60 splicing isoforms is critical for neural differentiation. These findings elucidate how alternative exons of a ubiquitously expressed splicing factor coordinate the temporal control of cell differentiation. PUF60 haploinsufficiency causes Verheij syndrome (VRJS), a multisystem developmental disorder characterized by intellectual disability, short stature, microcephaly, craniofacial dysmorphism, and cardiac, renal, and vertebral anomalies. Pathogenic variants include frameshift, missense, and splice-site mutations, often clustered within RRMs, leading to aberrant splicing and loss-of-function (Table S2, Figure 6).

Defects and mutations in PUF60 or CHD7 have been identified in human congenital disorders, specifically Verheij syndrome [76,77,78] and CHARGE syndrome [3,79,80,81]. CHARGE and Verheij syndromes are observed in newborns with congenital characteristics including ocular coloboma, heart defects, choanal atresia, growth and/or developmental retardation, genital and/or urinary abnormalities, and ear abnormalities and deafness [3,28,79,80,81]. *CHD7* is considered responsible for CHARGE syndrome, an autosomal dominant condition characterized by multiple congenital anomalies (Figure 5 and Figure 6). Both gain-of-function/dominant negative of FIR (overexpression of FIRΔexon2 in cancer) and loss-of-function of PUF60 (haploinsufficiency in VRJS) contribute to the pathology (Table S1, Figure 5). In various cancer types, FIRΔexon2 participates in the posttranscriptional regulation of brahma-related gene 1 (BRG1), an ATPase subunit of the SWItch/sucrose nonfermentable (SWI/SNF) chromatin remodeling complex [46]. Furthermore, BRG1 expression is affected by pre-mRNA splicing as a result of RNPs [82,83]. BRG1 controls multipotent neural crest formation together with CHD7 [84,85]. Integrative analysis identified codependent gene expression regulation by BRG1 and CHD7 at distal regulatory sites in embryonic stem cells [45]. Additionally, PUF60 has been reported to act as a chromatin remodeler alongside CHD7, facilitating the transcription of fibroblast growth factor 8 (FGF8) [46,85]. PUF60 cooperatively translocates from the nucleosome with CHD7 during neural development [85].

### 4.3. Spliceosomopathy

Spliceosome variants are responsible for human diseases known as spliceosomopathies [86]. Patients present with microcephaly, micrognathia, malar hypoplasia, external ear anomalies, ocular anomalies, psychomotor delay, intellectual disability, and defects of the limbs and heart, as mentioned above. The craniofacial malformations in these patients predominantly affect face and head structures derived from neural crest cells. Mutations in PUF60 are associated with craniofacial spliceosomopathies (Table S2, Figure 5) [87]. Recent reports of rare diseases such as Verheij syndrome patients with congenital anomalies harboring FIR/PUF60 mutations, also in CHARGE syndrome [74]. In this review, case reports have been summarized to provide an overview of mutations identified in genes associated with craniofacial spliceosomopathies due to splice site variants (Figure 6A). Mutations are found in the RRM1, RRM2, and RRM3/UHM regions (Figure 6B); while RRM1 is a particularly common site, its function needs to be revealed in the future.

### 4.4. Longevity and Immune Response

**Figure 7:**
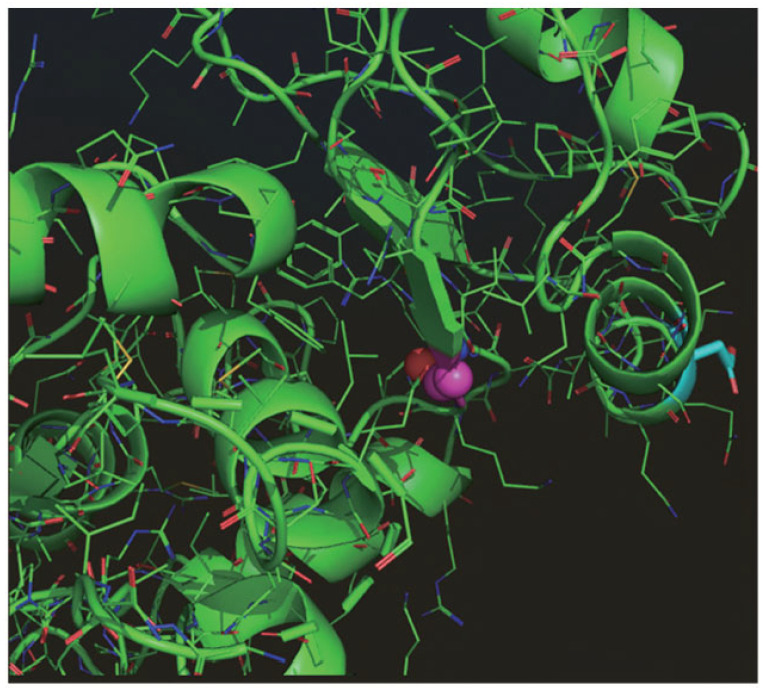
The glycine residue involved in rnp-6 (G281D) is located in the RRM2 motif. Gly281 is shown as a pink sphere within the FIRΔexon2 structure. The G281D mutation introduces a carboxylic acid side chain. This side chain extends into the solvent without clashing with other residues in the RRM2 motif. Therefore, the G281D mutation is presumed not to cause a conformational change in FIR. Instead, the G281D mutation is expected to disrupt RRM2 binding to other molecules, such as RNP. The pocket into which the carboxylic acid side chain projects is rich in positively charged polar residues, such as Lys and Arg. The presence of a negatively charged side chain will significantly interfere with molecular interactions in the pocket area (prepared by T.H).

Longevity. Although alterations in splicing fidelity are associated with the loss of homeostasis and aging, only a few splicing factors have been shown to be causally required to promote longevity, and the underlying mechanisms and downstream targets in these paradigms remain obscure. FIR/PUF60 shares structural homology with RNA-binding motif protein 39 (RBM39). RBM39 is involved in RNA splicing activation and transcription in cancer alongside FIR/PUF60. RNA-binding motif protein 39 (RBM39) has been reported as a promising therapeutic target for cancer [88]. Recently, it was reported that the ribonucleoprotein RNP-6/PUF60, a spliceosome component that promotes the recognition of weak 3′ splice sites, is involved in longevity [89]. In the nematode Caenorhabditis elegans, a gain-of-function mutation in rbm-39, a splicing factor that interacts with RNP-6/PUF60, caused aberrant splicing, enhanced stress responses, and extended lifespan [89]. A genetic suppressor screen revealed that this rbm-39 mutation increased nuclear speckle formation, alleviated splicing defects, and reduced the lifespan shortening caused by the RNP-6/PUF60 mutation. Using splicing changes induced by RNP-6/RBM-39 activity, we demonstrated that intron retention in EGL-8/phospholipase C β4 (PLCB4) is a critical splicing target that extends lifespan. Genetic and biochemical evidence suggests that neuronal RNP-6/EGL-8 controls organismal lifespan by suppressing mammalian target of rapamycin complex 1 (mTORC1) signaling. In mammalian cells, PUF60 suppression also potently and specifically inhibits mTORC1 signaling. These results demonstrate that splicing regulates lifespan via mTOR signaling. It is linked to autoimmune diseases (e.g., myositis) and viral/bacterial infections, likely through altered RNA processing and immune regulation. RBM39 is a key regulator of transcriptional networks via RNA splicing, DNA methylation and transcription, and nutrient sensing. RBM39 is a druggable metabolic sensor that links RNA splicing, transcriptional regulation, and metabolic reprogramming in cancer [90]. Splicing is a critical cellular process regulating important aspects of animal physiology, yet its role in controlling innate immunity is relatively unknown. PUF60 encodes an essential splicing factor that binds to polyuridine (poly(U)) tracts and promotes the association of the U2 small nuclear RNP (U2 snRNP) complex with primary transcripts. PUF60 is required for cell viability, proliferation, migration, and DNA damage repair in vitro [29,30]. In a previous genetic screen for *C. elegans* longevity regulators using cold tolerance as a proxy, we identified a novel mutation in the worm PUF60 ortholog, *rnp-6*, featuring a Gly281Asp substitution (hereafter referred to as *rnp-6(G281D)*) in the second RRM, which extends lifespan [90] (Figure 7).

Genetic screens using C. elegans reveal that the activity of the splicing factor RNP-6/PUF60 suppresses immunity while extending lifespan, suggesting a trade-off between these processes. Exposure to bacterial pathogens affects gene expression and splicing in an RNP-6/PUF60-dependent manner, and RNP-6/PUF60 gain- and loss-of-function activity reveals a positive role in immune regulation. Another longevity-promoting splicing factor, SFA-1, also exerts immunosuppressive effects, acting downstream of or in parallel with RNP-6/PUF60. RNP-6/PUF60 regulates immunity via TIR-1/PMK-1/MAPK signaling. The mammalian homolog, PUF60, also exhibits anti-inflammatory properties, and its levels decrease rapidly following bacterial infection in mammalian cells, suggesting a function in the host response. Together, these findings suggest the evolutionary conservation of immune regulation by specific components of the splicing machinery. FIR/PUF60 and RBM-39 are involved in immune responses [91].

## 5. Selection of a Specific Open Reading Frame (ORF) from Multiple Transcribed ORFs (ORF Dominance) in PUF60

**Figure 8:**
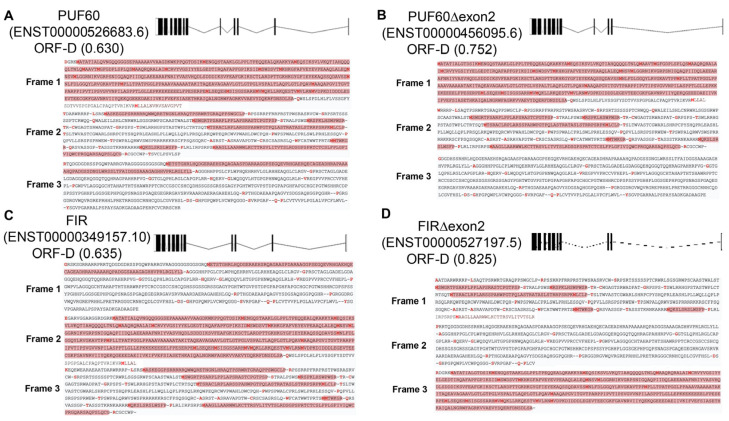
Coding and noncoding RNAs detected by dominant ORF analysis of PUF60 using ENSEMBL and RefSeq data. Red letters indicate the first amino acid residue following the stop codon. Red highlighting denotes potential ORFs that start with a methionine residue. Red letters indicated long coding RNA (mRNA) which means ORF dominance. When FIR transcripts were analyzed using ENSEMBL and RefSeq data, the ORF dominance scores were as follows: (**A**) PUF60 (ENSEMBL: 0.630/ RefSeq: 0.630), (**B**) PUF60Δexon2 (0.752/0.752), (**C**) FIR (0.635/0.635), and (**D**) FIRΔexon2 (0.825/0.746). These results indicate that among PUF60 RNA isoforms, the FIRΔexon2 transcript is likely to be translated most efficiently in the above available databases (Table S1). Regarding RNA isoforms of the *PUF60* gene and ORF dominance, three reading frames from 5′ to 3′ are shown. Sequences highlighted in red represent potential ORFs. Numerous secondary ORFs, in addition to the primary ORF, are observed in PUF60 and FIR, whereas they are reduced in PUF60Δexon2 and FIRΔexon2. ORFs were identified using the Expasy Translate tool. The transcribed RNA contains multiple ORFs, and the selection of the dominant ORF determines the expression of noncoding or coding RNAs—which are translated into proteins—and ultimately influences disease pathology (dominant ORF). Splicing is also believed to play a role in the dominant ORF RNAs involved in the expression of noncoding and coding RNAs.

Disease-specific diversity of RNA transcripts arises from factors such as alternative splicing, ribosomal abnormalities, and other regulatory mechanisms (Figure 5). Recently, it has been reported that when species face the risk of extinction, the expression patterns of RNA isoforms change, accompanied by an increase in ORF dominance, an index correlated with translational efficiency, thereby promoting the emergence of novel genes [22]. Interestingly, such an increase in ORF dominance is also observed during cancer initiation and progression [92]. Notably, noncoding RNAs that give rise to neoantigens have been shown to exhibit high ORF dominance, suggesting a link between ORF dominance and the generation of immunogenic peptides [92]. In particular, in neuroblastoma, the MYCN oncogene positively regulates and maintains the expression of RNA isoforms with high ORF dominance, and the expression of high ORF dominance transcripts in neuroblastoma is associated with poor patient prognosis [23]. In this context, ORF dominance calculations were performed for all PUF60 entries registered in two databases (RefSeq and ENSEMBL) [22,23]. These databases are constantly updated and include nearly all transcripts detected worldwide to date (with new data added daily). Therefore, the PUF60 data are not specific to a particular cell type or tissue but represent all transcripts detected to date (Table S1, Figure 3). Ribosomes bind to RNA and are thought to translate all three frames without discrimination. The longer translation time for physiologically relevant frames occurs when the RNA sequence has characteristics such as a lack of redundant ORFs (high ORF dominance), which is thought to correlate with translation efficiency. Coding RNAs share this characteristic with noncoding RNAs, and as they evolve from noncoding to coding RNA, ORF dominance increases. Conversely, RNAs that function as noncoding RNAs are more functional if they are translated by ribosomes more quickly, so ORF dominance is thought to gradually decrease over time. In the case of FIR, splicing mutations may affect the frames that are most likely to be translated, and FIRΔexon2 is expressed as a relatively high protein because the interfering frames are not read (Table S1, Figure 3). Information regarding the expression of each transcript in every cell or tissue is not necessarily available for all 21 transcript isoforms (Table S1). This is because most comprehensive expression analysis data to date rely on short-read RNA sequencing, which reflects the expression level of genes mapped to short reads rather than the expression level of individual RNA isoforms [22,23] (Table S1). This situation may change in the future if long-read RNA sequencing becomes commonplace. Recently, a novel metric termed ORF dominance was developed to quantitatively assess the coding potential of any RNA transcript, regardless of its classification as coding or noncoding [22]. ORF dominance is defined as the ratio of the length of the longest ORF in a transcript to the total length of all potential ORFs (Figure 8). Although conceptually simple, this index correlates with translation efficiency and has been applied to assess the translatability of noncoding RNAs, annotate new genes, and explore their relationships with evolution and cancer [22,23,92].

Major applications of this analytical approach include:

- Assessment of the translational capacity of noncoding RNAs
- Annotation of novel genes
- Integrated analysis with ribosome profiling
- Classification by ORF dominance score (coding vs. noncoding)
- Discovery of novel peptides and the functional analysis of disease-related noncoding RNAs.

Furthermore, analysis of coding and noncoding FIR transcripts revealed a considerable number of variants exhibiting a wide range of ORF dominance scores (Figure 3, Table S1). According to the ENSEMBL database, 21 FIR-related transcripts were identified, including the four aforementioned coding RNAs, whereas RefSeq data identified eight transcripts. Among the 21 ENSEMBL transcripts, 15 contained short ORFs ranging from 41 to 151 amino acids. Notably, five of these transcripts displayed ORF dominance scores exceeding 0.5, suggesting a potential coding capacity that warrants further experimental validation.

## 6. Diagnostic Biomarker and Therapeutic Potential

### 6.1. Biomarker

Autoantibodies against FIR and FIRΔexon2 have been detected in various types of cancer and autoimmune diseases [69,93], indicating that FIR and FIRΔexon2 proteins are expressed and recognized by the host immune system. Anti-FIRΔexon2 antibodies have been reported as a candidate biomarker common to ESCC and colorectal cancer (CRC). Specifically, it has been demonstrated that a combination of anti-p53, CEA, and anti-FIRΔexon2 antibodies improves diagnostic efficiency, thereby aiding in the early detection of ESCC and CRC. Furthermore, anti-FIRΔexon2 antibodies detected in patients with gastric cancer serve as a potential biomarker for monitoring favorable prognosis. However, further multi-institutional prospective studies are required to determine the sensitivity and specificity of this combined detection approach. Disease biomarkers (autoantibodies) for conditions other than cancer have been reported in psychiatric disorders (depression) [94], dermatomyositis [95,96], and Sjögren’s syndrome [91].

### 6.2. Cancer Gene Therapy

Targeting PUF60 may complement therapies for cancers harboring splicing factor mutations (e.g., SF3B1, U2AF1). Cancer treatment using FIR expression vectors (in animal models) [31,97]. Ad-FIR is involved in the DNA damage repair response induced by bleomycin (BLM) and is therefore applicable to other DNA-damaging agents. Ad-FIR increased BLM-induced DNA damage as indicated by γH2AX in vitro. Furthermore, Ad-FIRΔexon2 decreased Ku86 expression. The combination of Ad-FIR and BLM in the presence of TP53 siRNA increased DNA damage [98]. A nontransmissible, fusion gene-deficient Sendai virus (SeV) vector encoding FIR cDNA, SeV/dF/FIR, was prepared. The molecular mechanism of the antitumor effect and c-Myc suppression by SeV/dF/FIR was investigated using Spliceostatin A (SSA), an SF3B1 inhibitor, or SF3B1 siRNA, which induces c-Myc by increasing FIR∆exon2 in HeLa cells. Moreover, in nude mouse tumor xenograft models, SeV/dF/FIR exhibited high antitumor efficacy against human cancer cells. SeV/dF/FIR-mediated FIR expression showed high gene transduction efficiency with significant antitumor effects and apoptosis induction in HeLa and SW480 cells. SeV/dF/FIR demonstrated strong suppression of tumor growth without significant side effects in an animal xenograft model; therefore, SeV/dF/FIR is potentially applicable to future clinical cancer treatment [31]. SeV/ΔF vector-mediated FIR gene therapy demonstrated effective tumor suppression in HNSCC, suggesting that this therapy may have potential for clinical use as a novel strategy for the treatment of HNSCC [99] and pleural mesothelioma [100]. Additionally, airway-targeted gene therapy using the c-myc suppressor FIR via a recombinant SeV vector prevented tracheal stenosis in a rat model of airway mucosal injury [97].

### 6.3. A Low-Molecular-Weight Compound BK697

**Figure 9:**
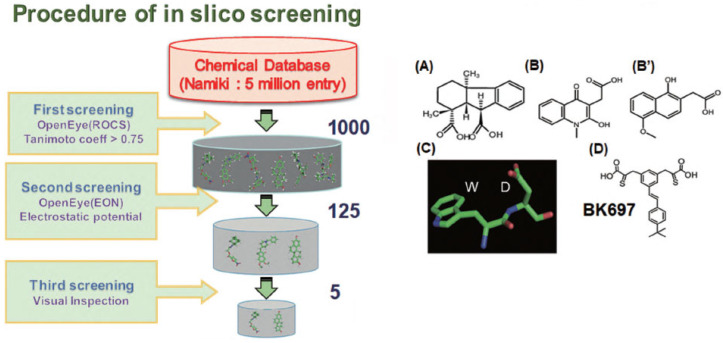
In silico screening procedure for the small-molecule chemical compound BK697 against FIRΔexon2. Small-molecule compounds binding to FIR (His-FIR, 645 μg/mL) and FIRΔexon2 (His-FIRΔexon2, 652 μg/mL) were screened using a natural product-based library consisting of 23,275 chemicals at the RIKEN NPDepo (Saitama, Japan) [42,43,46]. (**A**) A compound was identified as binding to FIRΔexon2. To find synthetic chemicals resembling the identified compound, in silico screening was performed using the Namiki chemical database containing 5 million entries. First, 1000 compounds were selected from the database based on chemical structural similarity. Second, the selected compounds were reduced to 125 based on charge distributions around the molecules. Finally, five candidates were selected by visual inspection. (**B**,**B’**) Two potent candidates selected from the screening. The chemical structure of these two candidates appears to mimic the Trp-Asp dipeptide in (**C**). (**D**) A compound derived from B, labeled BK697 (http://dx.doi.org/10.20517/2394-5079.2018.81) [46].

A screen of 23,275 chemical products from the Natural Product Depository (NPDepo) Array at RIKEN was conducted to identify small-molecule compounds targeting FIR and FIRΔexon2 [46,101,102,103]. Among these, BK697, which potentially interacts with FIRΔexon2 [46], significantly inhibited tumor cell growth by suppressing RP expression, indicating its potential as a therapeutic strategy in a preclinical study (Figure 9). BK697 represents a potential anticancer drug that suppresses rRNA and mRNA transcription by promoting the FIR-P62 interaction [5]. This study indicated that the c-myc-independent mechanism mediated by FIRΔexon2 contributes, at least in part, to the upregulation of rRNA and mRNA transcription in cancer cells. BK697 suppressed tumor cell growth [5,46]. A compound capable of binding to FIRΔexon2 was initially identified through the experimental screening of a natural product-based chemical library comprising 23,275 compounds (Figure 9A). BK697 was synthesized as an analog of inhibitory compounds identified by computational screening. Based on the identified natural product, two hit compounds were found via computational screening using a commercial chemical database provided by Namiki Inc. (Figure 9B,B’). Interestingly, these hit compounds structurally resemble the Trp-Asp dipeptide (Figure 9C). As nearly all compounds in the Namiki database are produced via organic synthesis, the two hit compounds are amenable to efficient derivative generation. Dozens of analogs were synthesized based on these hits. Among the analogs, BK697 exhibited the most promising activity for suppressing cancer cell growth as preclinical study.

Therapeutic targeting: A selective small-molecule inhibitor (SF2-69) targeting the UHM domain of PUF60 demonstrated antileukemic activity by inducing cell cycle arrest and modulating p53-dependent pathways. PUF60 inhibitors (e.g., SF2-69) are being investigated for hematologic malignancies [104].

### 6.4. Future Issues and Challenges to Be Addressed

The mechanisms involved in human diseases include splicing, rRNA-mRNA coupling, non-coding or coding RNA transcription and ORF-dominancy, and abnormalities in ribosome biogenesis. Although these are all interrelated, their relationship to disease and their details remain largely unknown, as described below.

(1) Elucidation of the common activation mechanism of rRNA and mRNA during neural differentiation and carcinogenesis. Simultaneous transcriptional activation of rRNA and mRNA in cancer cells requires a rapid increase in protein synthesis capacity necessary for cell proliferation, suggesting coordinated regulation of nucleolar function and RNA polymerase activity.
  - Activation of the mTOR signaling pathway
  - Overexpression of MYC
  - Epigenetic chromatin remodeling
(2) FIR/PUF60 is involved in cancer cell proliferation, metabolism, and survival through regulation of RNA splicing and mRNA stability. Meanwhile, mTOR signaling functions as an upstream regulator of translation, metabolism, and the cell cycle. Future research will clarify whether PUF60 alters splicing patterns depending on the activation state of the mTOR pathway or whether mTOR inhibition affects PUF60 function. The hypothesis that FIR/PUF60 regulates translation efficiency through splicing control is an intriguing topic for elucidating the molecular mechanisms of cancer and metabolic diseases.
(3) How is unified transcription of human rDNA possible? What is the connection between ribosomal abnormalities and splicing abnormalities?
  - Shared and Interfered Nuclear Structures: Ribosome biogenesis occurs in the nucleolus, and splicing occurs in nuclear speckles. Reorganization of the nuclear structure due to nucleolar stress may also affect the localization and function of splicing factors.
  - Multifunctionality of Ribosomal Proteins
  - Coordinated Regulation of Translation and Splicing: Recent studies have suggested that translational regulation and splicing regulation are linked, and that ribosomal abnormalities may affect the selection of mRNA splice variants. In particular, the stress response associated with ribosomal pathologies can lead to changes in the expression and modification of splicing factors.
(4) Main Reasons Why Abnormal Splicing Causes Ribosomal Abnormalities
  - Abnormal mRNA Splicing of Ribosomal Components. Abnormal splicing of pre-mRNAs encoding components such as ribosomal proteins (RPs) and rRNA-modifying enzymes results in a decrease in translatable mRNAs or the accumulation of abnormal isoforms.
  - Impaired rRNA Processing Due to Abnormal snRNP Function. Mutations in spliceosome components (e.g., SF3B1, U2AF1) also affect pre-rRNA processing through snRNP dysfunction. Accurate cleavage and modification of rRNA are inhibited, resulting in incomplete formation of mature ribosomes.
  - Disrupted Nucleolar Function. Abnormal splicing alters the expression and localization of nucleolar-localized proteins. Because the nucleolus is the site of rRNA transcription, processing, and ribosome assembly, changes in nucleolar structure are directly linked to ribosome formation.
  - Decreased rDNA Transcription Due to Impaired Transcription Elongation. rDNA transcription is Pol I-dependent, but it has been reported that intranuclear crosstalk also suppresses Pol I activity, resulting in reduced rRNA production. Investigating the involvement of FIR/PUF60 splicing in these mechanisms is a future challenge that will provide new insights into the mechanisms of neuronal differentiation and oncogenesis.

### 6.5. Limitations

(1) The mechanism and the specific d proteins described in this review are limited and require further identification.
(2) The interprepation among intermolecular crosstalk is complicated and not well supported by current knowledge:
  - Abnormal splicing → Abnormal mRNA → Abnormal translation → Ribosomal stress.
  - Ribosomal abnormalities → Altered translation selectivity → Altered expression and modification of splicing factors.
  - Functional interference due to rearrangements of intranuclear structures (nucleoli and speckles).

## 7. Discussion

This review surveyed the link between transcription and splicing, or co-regulation of mRNA and rRNA in human diseases in terms of FIR/PUF60 multifunction model. Germline variant of *FIR/PUF60* causes developmental disorders with brain malignant tumor is described in the literature [105] (Patient 4 was diagnosed with pineoblastoma (WHO Grade IV), c1574T>A, pVal525Glu). Specifically, this review tried to explain the pathogenesis of human diseases, via RNA analysis, how FIR/PUF60 splicing participates in the coordinated control of mRNA and rRNA expression and various noncoding and coding RNAs are transcribed from the *FIR/PUF60* gene [5]. The mechanisms underlying the diversification of proteins expressed in cells are complex and involve transcription, splicing, interactions between noncoding and coding RNAs, and epigenetics. In cancer cells, the simultaneous transcriptional activation of rRNA and mRNA is driven by the need for a rapid increase in protein synthesis capacity required for cell proliferation. This involves the coordinated regulation of nucleolar function and RNA polymerase activity.

PUF60/FIR is involved in oncogenesis and neurodevelopment through its splicing function and c-Myc expression (Figure 5). In this review, we focused and outlined the diverse functions of PUF60/FIR as a c-myc-dependent mechanism [5]; however, c-myc-independent role of FIRΔexon2 in regulating transcription, RNA splicing, rRNA/mRNA coregulation is intriguing but not always clearly separated from c-myc-dependent mechanisms.

It was shown that multiple RNAs transcribed by aberrant splicing exist as RNAs of various lengths via three different ORFs (Figure 8, Table S1). Even short RNAs generated by splicing and ORF selection interact with each other to affect cellular functions, potentially contributing to neuronal differentiation and oncogenesis. RNA translation in ribosomes is even more complex because ribosomal proteins themselves change in disease (Figure 3 and Figure 5). These findings suggest that the complex interactions of splicing, ORF dominance, and ribosomal protein aberrations may be involved in the heterogeneity of cancer cells and the evolution of life. Our results suggest that FIRΔexon2 mediates a novel cancer-related mRNA and rRNA transcription coactivation pathway via TFIIH/P62 [5]. Furthermore, we investigated low-molecular-weight compounds that bind to FIR and FIRΔexon2 as potential therapeutic agents for cancer. The low-molecular-weight compound BK697, which interacts with FIRΔexon2, inhibited tumor cell proliferation by suppressing rRNA expression [5,46]. It is expected that low-molecular-weight compounds inhibiting FIRΔexon2 expression will act as cell growth inhibitors via a novel mechanism that disrupts the transcriptional activation chain between mRNA and rRNA in cancer cells (Figure 2A). This challenging research is highly significant for the development of low-cost drugs. A novel function for FIR or FIRΔexon2 was proposed using comprehensive RNA sequencing and ChIP-seq analysis of gene expression across transcription, alternative splicing, and histone modifications in position-specific genomic regions [5,12]. This new function of the *FIR (PUF60)* gene is derived from the integration of histone modification, transcription, alternative splicing, and especially RiBi, potentially serving as a missing link between carcinogenesis and congenital disease [5,12]. We discuss how the FIR family acts as multifunctional proteins in gene expression dynamics, not only in cancers but also in neural development, in the context of RiBi via MYC-dependent and independent pathways. FIRΔexon2 competed with FIR for P62 binding and coactivated mRNA and rRNA transcription. Small-molecule compounds that bind to FIR and FIRΔexon2 were screened for potential cancer therapies (Figure 2B,C) [5,12]. The small-molecule compound BK697, which interacts with FIRΔexon2, inhibited tumor cell proliferation and suppressed rRNA expression. This study proposed a novel cancer-related coactivation pathway for mRNA and rRNA transcription mediated by FIRΔexon2 via TFIIH/P62 (Figure 2B,C) [5]. Future studies will require direct evidence from X-ray crystallography to elucidate the structural differences between FIR and FIRΔexon2 that affect the P62-RBP6 interaction. The small-molecule compounds introduced in this study, which inhibit FIRΔexon2 expression, are expected to act as cell growth inhibitors via a novel mechanism that disrupts the mRNA and rRNA transcriptional activation chain in cancer cells (Figure 2B,C).

As another topic, coding and non-coding RNA synthetic mechanisms and aberrant splicing events resulting from alterations in open reading frame (ORF) dominance (Figure 8). The consequent shifts in translation efficiency are strongly implicated in cancer pathogenesis [23,99] and, potentially, neurodevelopmental disorders [22]. These abnormalities contribute to disease through dysregulated protein expression and the disruption of cell cycle control. Shifts in ORF dominance led to the preferential translation of specific ORFs as a result of aberrant mRNA splicing. These events can suppress the translation of canonical ORFs, thereby promoting the production of abnormal proteins. Splicing is tightly regulated during neurodevelopment; therefore, disruptions related to ORF dominance can alter the expression of proteins essential for neuronal differentiation, migration, and synaptogenesis. Consistent with this concept, several human noncoding RNAs exhibiting high ORF dominance are predicted to participate in brain development and tumorigenesis [22]. In neuroblastoma, MYCN-regulated genes with high ORF dominance predominantly encode components of the splicing machinery, the spliceosome, and the translational apparatus itself [23]. In cancer, recurrent mutations in splicing factors such as SF3B1, SRSF2, and U2AF1 are frequently observed [106], and these aberrations likely induce translational dysregulation associated with ORF dominance (Figure 8) [92].

Splicing homeostasis is dynamically altered in diverse contexts, including oncogenesis, neurodevelopment, lifespan, and immune response. Recently, splicing factors such as PUF60 (FIR) and RBM-39 have been shown to play important roles in oncogenesis, neurodevelopment, lifespan, and immune response via mTORC1 signaling. In this study, we investigated PUF60 (FIR) expression and its influence on the mTORC1 signaling pathway. This review manuscript focuses on aberrant splicing and presents promising new findings on disease-related protein diversity of *FIR/PUF60*, novel cell signaling targets, and mechanisms for effective individualized therapies in human diseases that may lead to future diagnostic and therapeutic applications.

## Data Availability

The RNA-seq data in this study is deposited in GEO data base available as GSE206465 (GSM6254981-GSM6254994). BK697 is available by requesting to K.M. Correspondence and requests for materials should be addressed to K.M.

## Supplementary Materials

The following supporting information can be downloaded at: https://www.mdpi.com/article/10.3390/ijms27020643/s1.
