# Supplementary material for: FIR/PUF60: Multifunctional Molecule Through RNA Splicing for Revealing the Novel Disease Mechanism and Effective Individualized Therapies
Source: Int J Mol Sci. 2026 Jan 8;27(2):643. doi: 10.3390/ijms27020643 (PMC12841381; doi:10.3390/ijms27020643)
Supplement: Supplementary file 1 [file ijms-27-00643-s001.zip › Table S1.pdf]

Supplementary Table 1. Coding and noncoding RNAs detected by dominant ORF analysis of PUF60 using ENSEMBL and RefSeq data.

#ID	#Genename	#length_of_transcript	#longestORF	#ORF dominance
ENST00000526151	cdna chromosome:GRCh38:8:143821349:143829315:-1 gene:ENSG00000179950.14 gene_biotype:protein_coding transcript_biotype:retained_intron gene_symbol:PUF60 description:poly(U) binding splicing factor 60 [Source:HGNC Syr	557	151	0.674107
ENST00000532127	cdna chromosome:GRCh38:8:143820426:143829319:-1 gene:ENSG00000179950.14 gene_biotype:protein_coding transcript_biotype:retained_intron gene_symbol:PUF60 description:poly(U) binding splicing factor 60 [Source:HGNC Syr	604	129	0.381657
ENST00000531995	cdna chromosome:GRCh38:8:143820233:143821743:-1 gene:ENSG00000179950.14 gene_biotype:protein_coding transcript_biotype:retained_intron gene_symbol:PUF60 description:poly(U) binding splicing factor 60 [Source:HGNC Syr	631	74	0.496644
ENST00000527584	cdna chromosome:GRCh38:8:143818373:143824460:-1 gene:ENSG00000179950.14 gene_biotype:protein_coding transcript_biotype:retained_intron gene_symbol:PUF60 description:poly(U) binding splicing factor 60 [Source:HGNC Syr	547	106	0.579235
ENST00000533162	cdna chromosome:GRCh38:8:143818222:143829338:-1 gene:ENSG00000179950.14 gene_biotype:protein_coding transcript_biotype:protein_coding gene_symbol:PUF60 description:poly(U) binding splicing factor 60 [Source:HGNC Syr	761	106	0.430894
ENST00000529693	cdna chromosome:GRCh38:8:143818193:143818963:-1 gene:ENSG00000179950.14 gene_biotype:protein_coding transcript_biotype:retained_intron gene_symbol:PUF60 description:poly(U) binding splicing factor 60 [Source:HGNC Syr	684	85	0.377778
ENST00000531897	cdna chromosome:GRCh38:8:143817996:143829304:-1 gene:ENSG00000179950.14 gene_biotype:protein_coding transcript_biotype:protein_coding gene_symbol:PUF60 description:poly(U) binding splicing factor 60 [Source:HGNC Syr	785	89	0.388646
ENST00000528999	cdna chromosome:GRCh38:8:143817979:143819037:-1 gene:ENSG00000179950.14 gene_biotype:protein_coding transcript_biotype:processed_transcript gene_symbol:PUF60 description:poly(U) binding splicing factor 60 [Source:HGN	431	29	0.690476
ENST00000529999	cdna chromosome:GRCh38:8:143817950:143829044:-1 gene:ENSG00000179950.14 gene_biotype:protein_coding transcript_biotype:protein_coding gene_symbol:PUF60 description:poly(U) binding splicing factor 60 [Source:HGNC Syr	867	89	0.419811
ENST00000528320	cdna chromosome:GRCh38:8:143817886:143824399:-1 gene:ENSG00000179950.14 gene_biotype:protein_coding transcript_biotype:retained_intron gene_symbol:PUF60 description:poly(U) binding splicing factor 60 [Source:HGNC Syr	922	131	0.422581
ENST00000531951	cdna chromosome:GRCh38:8:143817866:143829028:-1 gene:ENSG00000179950.14 gene_biotype:protein_coding transcript_biotype:processed_transcript gene_symbol:PUF60 description:poly(U) binding splicing factor 60 [Source:HGN	973	106	0.540816
ENST00000527744	cdna chromosome:GRCh38:8:143817586:143829299:-1 gene:ENSG00000179950.14 gene_biotype:protein_coding transcript_biotype:protein_coding gene_symbol:PUF60 description:poly(U) binding splicing factor 60 [Source:HGNC Syr	1007	106	0.436214
ENST00000526459	cdna chromosome:GRCh38:8:143817578:143829338:-1 gene:ENSG00000179950.14 gene_biotype:protein_coding transcript_biotype:protein_coding gene_symbol:PUF60 description:poly(U) binding splicing factor 60 [Source:HGNC Syr	1003	89	0.393805
ENST00000532884	cdna chromosome:GRCh38:8:143817080:143818476:-1 gene:ENSG00000179950.14 gene_biotype:protein_coding transcript_biotype:protein_coding gene_symbol:PUF60 description:poly(U) binding splicing factor 60 [Source:HGNC Syr	819	47	0.52809
ENST00000524570	cdna chromosome:GRCh38:8:143817042:143823282:-1 gene:ENSG00000179950.14 gene_biotype:protein_coding transcript_biotype:processed_transcript gene_symbol:PUF60 description:poly(U) binding splicing factor 60 [Source:HGN	1934	47	0.333333
ENST00000527197	cdna chromosome:GRCh38:8:143816520:143829320:-1 gene:ENSG00000179950.14 gene_biotype:protein_coding transcript_biotype:protein_coding gene_symbol:PUF60 description:poly(U) binding splicing factor 60 [Source:HGNC Syr	1559	513	0.824759
ENST00000349157	cdna chromosome:GRCh38:8:143816377:143829352:-1 gene:ENSG00000179950.14 gene_biotype:protein_coding transcript_biotype:protein_coding gene_symbol:PUF60 description:poly(U) binding splicing factor 60 [Source:HGNC Syr	1821	542	0.63466
ENST00000456095	cdna chromosome:GRCh38:8:143816377:143829303:-1 gene:ENSG00000179950.14 gene_biotype:protein_coding transcript_biotype:protein_coding gene_symbol:PUF60 description:poly(U) binding splicing factor 60 [Source:HGNC Syr	1736	530	0.751773
ENST00000313352	cdna chromosome:GRCh38:8:143816377:143829039:-1 gene:ENSG00000179950.14 gene_biotype:protein_coding transcript_biotype:protein_coding gene_symbol:PUF60 description:poly(U) binding splicing factor 60 [Source:HGNC Syr	1804	499	0.61529
ENST00000453551	cdna chromosome:GRCh38:8:143816377:143829026:-1 gene:ENSG00000179950.14 gene_biotype:protein_coding transcript_biotype:protein_coding gene_symbol:PUF60 description:poly(U) binding splicing factor 60 [Source:HGNC Syr	1842	516	0.610651
ENST00000526683	cdna chromosome:GRCh38:8:143816344:143829315:-1 gene:ENSG00000179950.14 gene_biotype:protein_coding transcript_biotype:protein_coding gene_symbol:PUF60 description:poly(U) binding splicing factor 60 [Source:HGNC Syr	1868	559	0.629505
ref|NM_001271100	Homo sapiens poly(U) binding splicing factor 60 (PUF60), transcript variant 8, mRNA	1916	499	0.61529
ref|NM_001271099	Homo sapiens poly(U) binding splicing factor 60 (PUF60), transcript variant 7, mRNA	1861	530	0.751773
ref|NM_001271098	Homo sapiens poly(U) binding splicing factor 60 (PUF60), transcript variant 6, mRNA	1945	558	0.629087
ref|NM_001271097	Homo sapiens poly(U) binding splicing factor 60 (PUF60), transcript variant 5, mRNA	1810	513	0.74564
ref|NM_001271096	Homo sapiens poly(U) binding splicing factor 60 (PUF60), transcript variant 4, mRNA	1894	541	0.634232
ref|NM_001136033	Homo sapiens poly(U) binding splicing factor 60 (PUF60), transcript variant 3, mRNA	1967	516	0.610651
ref|NM_014281.4|	Homo sapiens poly(U) binding splicing factor 60 (PUF60), transcript variant 2, mRNA	1897	542	0.63466
ref|NM_078480.2|	Homo sapiens poly(U) binding splicing factor 60 (PUF60), transcript variant 1, mRNA	1948	559	0.629505
