# Supplementary material for: FIR/PUF60: Multifunctional Molecule Through RNA Splicing for Revealing the Novel Disease Mechanism and Effective Individualized Therapies
Source: Int J Mol Sci. 2026 Jan 8;27(2):643. doi: 10.3390/ijms27020643 (PMC12841381; doi:10.3390/ijms27020643)
Supplement: Supplementary file 1 [file ijms-27-00643-s001.zip › Table S2.pdf]

Table S2. Human germline *PUF60* mutation, delition, and duplication list and reference in the literature.

No.	8q24 deletion and duplication	cDNA variant	Protein alteration	Effect	Reference	DOI
1	8q24 deletion (8.35Mbp) including PUF60.	-	-	-	Verheij J B G M et al. An 8.35 Mb overlapping interstitial deletion of 8q24 in two patients with coloboma, congenital heart defect, limb abnormalities, psychomotor retardation and convulsions. Eur. J. Med. Genet. 52, 353–357 (2009).	https://doi.org/10.1016/j.ejmg.2009.05.006
2	8q24.3 deletion (78kb-1Mbp) including PUF60. (n=5)	c.505C>T	p.His169Tyr	Missense	Dauber A. et al. SCRIB and PUF60 are primary drivers of the multisystemic phenotypes of the 8q24.3 copy-number variant. Am. J. Hum. Genet. 93, 798–811 (2013).	https://doi.org/10.1016/j.ajhg.2013.09.010
3	8q24.3 deletion (1Mbp) including PUF60.	-	-	-	Wells C, Spaggiari E, Malan V et al: First fetal case of the 8q24.3 contiguous genes syndrome. Am J Med Genet 2016; 170: 239–242.	
4	8q24 deletion (13.1kbp) including PUF60.	-	-	-	Abdin D. et al. PUF60–SCRIB fusion transcript in a patient with 8q24.3 microdeletion and atypical Verheij syndrome. Eur. J. Med. Genet. 62, 103587 (2019).	https://doi.org/10.1016/j.ejmg.2018.11.021
5	8q24 duplication (4.6Mbp) including PUF60.	-	-	-	Yue F. et al. Molecular cytogenetic characterization of 1q42.3q44 deletion and 8q24.3 duplication in a fetus with single umbilical artery and ventricular septal defects. Taiwan J. Obstet. Gynecol. 60, 1126–1133 (2021).	https://doi.org/10.1016/j.tjog.2021.09.030
6	-	c.24+1G>C<br>c.1342C>T<br>c.1448T>C<br>c.407_410delTCTA<br>c.901A>T	 p.Arg448*<br>p.Val483Ala<br>p.Ile136Thrfs*31<br>p.Lys301*	Splice variant (splice donor)<br>Nonsense<br>Missense<br>Frameshift<br>Nonsense	El Chehadeh S. et al. Dominant variants in the splicing factor PUF60 cause a recognizable syndrome with intellectual disability, heart defects and short stature. Eur. J. Hum. Genet. 25, 43–51 (2016).	https://doi.org/10.1038/ejhg.2016.133
7	-	c.541G>A<br>c.439C>T<br>c.1144+1G>A	p.Glu181Lys<br>p.Gln147* 	Missense<br>Nonsense<br>Splice variant (splice donor)	Santos-Simarro F. et al. Eye coloboma and complex cardiac malformations belong to the clinical spectrum of PUF60 variants. Clin. Genet. 92, 350–351 (2017).	https://doi.org/10.1111/cge.12965
8	-	c.931_932insAAAA<br>c.604-2A>G<br>c.541G>A<br>c1472G>A<br>c.1577_1587del<br>c.475G>A<br>c.1008+1G>A<br>c.604-2A>C<br>c.1094dupG<br>c.1381-2A>G<br>c.619_637del<br>c24+1G>A	p.Thr311Lysfs*13<br><br>p.Glu181Lys<br>p.Gly491Glu<br>p.His526Profs*16<br>p.Asp159Asn<br><br><br>p.Thr366Hisfs*81<br><br>p.Asn207Profs*3 	Frameshift<br>Splice variant (splice acceptor)<br>Missense<br>Missense<br>Frameshift<br>Missense<br>Splice variant (splice donor)<br>Splice variant (splice acceptor)<br>Frameshift<br>Splice variant (splice acceptor)<br>Frameshift<br>Splice variant (splice donor)	Low K.J. et al. PUF60 variants cause a syndrome of ID, short stature, microcephaly, coloboma, craniofacial, cardiac, renal and spinal features. Eur. J. Hum. Genet. 25, 552–559 (2017).	https://doi.org/10.1038/ejhg.2017.27
9	-	c.698_699del	p.Val233Alafs*8	Frameshift	Zhao J.J. et al. Exome sequencing reveals NAA15 and PUF60 as candidate genes associated with intellectual disability. Am. J. Med. Genet. B Neuropsychiatr. Genet. 177, 10–20 (2018).	https://doi.org/10.1002/ajmg.b.32574
10	-	c.389G >A	p.Arg130His	Missense	Moccia A. et al. Genetic analysis of CHARGE syndrome identifies overlapping molecular biology. Genet. Med. 20, 1022–1029 (2018).	https://doi.org/10.1038/gim.2017.233
11	-	c.1357C > T	p.Gln453*	Nonsense	Xu Q. et al. Role of PUF60 gene in Verheij syndrome: a case report of the first Chinese Han patient with a de novo pathogenic variant and review of the literature. BMC Med. Genomics 11, 92 (2018).	https://doi.org/10.1186/s12920-018-0421-3
12	-	c.602A>C	p.Lys201Thr	Missense	Alkhunaizi E. et al. Clinical characterization of a PUF60 variant in a patient with Dubowitz-like syndrome. Am. J. Med. Genet. A 179, 130–133 (2019).	https://doi.org/10.1002/ajmg.a.60691
13	-	c.1673_1674del	p.Ser558Cysfs*21	Frameshift	Yamada M. et al. Protein elongation variant of PUF60: Milder phenotypic end of the Verheij syndrome. Am. J. Med. Genet. A 182, 2709–2714 (2020).	https://doi.org/10.1002/ajmg.a.61816
14	-	c.752dup	p.Gln252Profs*152	Frameshift	Haug P. et al. Whole exome sequencing in coloboma/microphthalmia: Identification of novel and recurrent variants in seven genes. Genes (Basel) 12, 65 (2021).	https://doi.org/10.3390/genes12010065
15	-	c.1008+1G>T<br>c.1452_1454del	 p.Glu486del	Splice variant (splice donor)<br>Deletion	Latypova X. et al. Letter regarding the article "two girls with short stature, short neck, vertebral anomalies, Sprengel deformity and intellectual disability" (Isidor et al., 2015). Eur. J. Med. Genet. 64, 104179 (2021).	https://doi.org/10.1016/j.ejmg.2021.104179
16	-	c1604G>C	p.Arg535Pro	Missense	Toader D.O. et al. Identification of a new variant of PUF60 gene: Case presentation and literature review. Cancer Diagn. Progn. 1, 213–219 (2021).	https://doi.org/10.21873/cdp.10029
17	-	c.1154_1166del<br>c.436C>T<br>c.803_809delGCTACGG<br>c.449_457delCCCCCTTTG<br>c.860dup<br>c.1459T>C<br>c.853dup<br>c.848_849del<br>c.1471G>A<br>c.382_383delAT	p.Pro385Leufs*12<br>p.Arg146Cys<br>p.Gly268Alafs*18<br>p.Ala150_Phe152del<br>p.Met287Ilefs*5<br>p.Cys487Arg<br>p.Ser285Phefs*7<br>p.Ala283Glyfs*8<br>p.Gly491Arg<br>p.Met128Valfs*11	Frameshift<br>Missense<br>Frameshift<br>Deletion<br>Frameshift<br>Missense<br>Frameshift<br>Frameshift<br>Missense<br>Frameshift	Fennell A.P. et al. The diverse pleiotropic effects of spliceosomal protein PUF60: A case series of Verheij syndrome. Am. J. Med. Genet. A 188, 3432–3447 (2022).	https://doi.org/10.1002/ajmg.a.62950
18	-	c.1594del<br>c.238_264del<br>c.850dup<br>c.1574T>A<br>c.449_457del<br>c.658G>T<br>c.818-20A>G<br>c.817+1G>T<br>c.424G>T<br>c.832_833del	p.Leu532Serfs*68<br>p.Lys80_Ile88del<br>p.Val284Glyfs*8<br>p.Val525Glu<br>p.Ala150_Phe152del<br>p.Glu220*<br><br><br>p.Glu142*<br>p.Gln278Valfs*13	Frameshift<br>Deletion<br>Frameshift<br>Missense<br>Deletion<br>Nonsense<br>Splice variant (splice acceptor)<br>Splice variant (splice donor)<br>Nonsense<br>Frameshift	Grimes H. et al. PUF60-related developmental disorder: A case series and phenotypic analysis of 10 additional patients with monoallelic PUF60 variants. Am. J. Med. Genet. A 191, 2610–2622 (2023).	https://doi.org/10.1002/ajmg.a.63313
19	-	c.1678T>A	p.*560Argext*204	Extention	Miao M. et al. Identification of a novel de novo PUF60 variant causing Verheij syndrome in a fetus. Gene 897, 148092 (2024).	https://doi.org/10.1016/j.gene.2023.148092
20	-	c.688del	p.Val230Trpfs*58	Frameshift	Hoogenboom A. et al. Novel PUF60 variant suggesting an interaction between Verheij and Cornelia de Lange syndrome: phenotype description and review of the literature. Eur. J. Hum. Genet. 32, 435–439 (2024).	https://doi.org/10.1038/s41431-023-01527-1
21	-	c.1179del	p.Ile394Serfs*7	Frameshift	Yacobi Bach M. et al. PUF60 loss-of-function with normal cognition should be considered in the differential diagnosis of Klippel-Feil syndrome. Am. J. Med. Genet. A 194, e63550 (2024).	https://doi.org/10.1002/ajmg.a.63550
22	-	c.206del<br>c.1658A>G<br>c.636_640del<br>c.1123C>T<br>c.510+1G>T	p.Lys69Argfs*16<br>p.Asp553Gly<br>p.Gln212Hisfs*7<br>p.Gln375* 	Frameshift<br>Missense<br>Frameshift<br>Nonsense<br>Splice variant (splice donor)	Baum E. et al. Novel genetic and phenotypic expansion in ameliorated PUF60-related disorders. Int. J. Mol. Sci. 25, 2053 (2024).	https://doi.org/10.3390/ijms25042053
23	-	c.713C>G	p.Ser238*	Nonsense	Ogawa T. et al. Identification of a de novo PUF60 variant associated with craniofacial microsomia. Am. J. Med. Genet. A 194, e63631 (2024).	https://doi.org/10.1002/ajmg.a.63631
24	-	c.382_383del	p.Met128Valfs*11	Frameshift	Mio C. et al. A prenatal diagnosis of Verheij syndrome in a fetus harboring a de novo PUF60 variant. Clin. Case Rep. 13, e71230 (2025).	https://doi.org/10.1002/ccr3.71230
